# Reviewing the consistency of Dissociative Identity Disorder: a case report.

**DOI:** 10.1192/j.eurpsy.2023.2061

**Published:** 2023-07-19

**Authors:** E. Herrero Pellón, P. Albarracín Marcos, M. Huete Naval, R. Galerón Guzmán, F. Mayor Sanabria, A. Montes Montero

**Affiliations:** 1 Hospital Clínico San Carlos, Madrid, Spain

## Abstract

**Introduction:**

We present the case of a 22-year-old patient who has been followed up in a daytime hospital for personality disorders since June 2022. Of note is the presence of multiple personalities (in total of more than 20 have been identified), each of which has distinct physical and psychological characteristics.

**Objectives:**

The objective is to present a clinical case of dissociative identity disorder and to review the existence of scientific evidence supporting this diagnosis.

**Methods:**

Literature review of scientific papers over the last years and classic textbooks on the issue. We included references in English and Spanish languages.

**Results:**

Numerous studies support that dissociative disorders are the result of psychological traumas that generally begin in childhood. This is a difficult category to diagnose, since they present symptoms that also appear in other disorders such as those of the schizophrenic spectrum.

One or more dissociative parts of the subject’s personality avoid the traumatic memories while others become fixed to these traumatic experiences and manifest symptoms. In the case of our patient, there are dissociative episodes with subsequent amnesia and auditory, visual and olfactory hallucinations, as well as impulsive behaviors in the form of self-injury and a flattened affect, with significant emotional distancing.

**Conclusions:**

- The prevalence of dissociative identity disorder is higher than traditionally thought.
- Some theories develop how trauma essentially produces a degree of dissociation of the psychobiological systems that constitute the subject’s personality.

**Disclosure of Interest:**

None Declared

